# Prevalence of chronic comorbidities in dengue fever and West Nile virus: A systematic review and meta-analysis

**DOI:** 10.1371/journal.pone.0200200

**Published:** 2018-07-10

**Authors:** Alaa Badawi, Russanthy Velummailum, Seung Gwan Ryoo, Arrani Senthinathan, Sahar Yaghoubi, Denitsa Vasileva, Emma Ostermeier, Mikayla Plishka, Marcel Soosaipillai, Paul Arora

**Affiliations:** 1 Public Health Risk Sciences Division, Public Health Agency of Canada, Toronto, ON, Canada; 2 Department of Nutritional Sciences, Faculty of Medicine, University of Toronto, Toronto, ON, Canada; 3 Faculty of Arts and Science, University of Toronto, Toronto, ON, Canada; 4 Mount Sinai Health System, Toronto, ON, Canada; 5 Faculty of Science, Ryerson University, Toronto, ON, Canada; 6 Faculty of Science, University of Waterloo, Waterloo, Waterloo, ON, Canada; 7 National Microbiology Laboratory, Infectious Disease Prevention and Control Branch, Public Health Agency of Canada, Toronto, ON, Canada; CEA, FRANCE

## Abstract

**Background:**

Flavivirus diseases such as dengue fever (DENV), West Nile virus (WNV), Zika and yellow fever represent a substantial global public health concern. Preexisting chronic conditions such as cardiovascular diseases, diabetes, obesity, and asthma were thought to predict risk of progression to severe infections.

**Objective:**

We aimed to quantify the frequency of chronic comorbidities in flavivirus diseases to provide an estimate for their prevalence in severe and non-severe infections and examine whether chronic diseases contribute to the increased risk of severe viral expression.

**Methods:**

We conducted a comprehensive search in PubMed, Ovid MEDLINE(R), Embase and Embase Classic and grey literature databases to identify studies reporting prevalence estimates of comorbidities in flavivirus diseases. Study quality was assessed with the risk of bias tool. Age-adjusted odds ratios (ORs) were estimated for severe infection in the presence of chronic comorbidities.

**Results:**

We identified 65 studies as eligible for inclusion for DENV (47 studies) and WNV (18 studies). Obesity and overweight (i.e., BMI> 25 kg/m^2^, prevalence: 24.5%, 95% CI: 18.6–31.6%), hypertension (17.1%, 13.3–21.8%) and diabetes (13.3%, 9.3–18.8%) were the most prevalent comorbidities in DENV. However, hypertension (45.0%, 39.1–51.0%), diabetes (24.7%, 20.2–29.8%) and heart diseases (25.6%, 19.5–32.7%) were the most prevalent in WNV. ORs of severe flavivirus diseases were about 2 to 4 in infected patients with comorbidities such as diabetes, hypertension and heart diseases. The small number of studies in JEV, YFV and Zika did not permit estimating the prevalence of comorbidities in these infections.

**Conclusion:**

Higher prevalence of chronic comorbidities was found in severe cases of flavivirus diseases compared to non-severe cases. Findings of the present study may guide public health practitioners and clinicians to evaluate infection severity based on the presence of comorbidity, a critical public health measure that may avert severe disease outcome given the current dearth of clear prevention practices for some flavivirus diseases.

## Introduction

The *Flaviviridae* is a large family of positive-strand RNA viruses, that comprises four genera: *Flavivirus*, *Pegivirus*, *Pestivirus*, and *Hepacivirus* [1]. The *Flavivirus* genus consists of more than 70 viruses, many of which are arthropod-borne human pathogens that cause a variety of clinical diseases, ranging from asymptomatic to mild fever to more severe diseases including encephalitis and hemorrhagic fever [1, 2] Most flaviviruses are transmitted through the bite of an infected arthropod vector, mainly *Aedes* genus (*Aedes aegypti* and to a lesser extent, *Aedes albopictus*) and *Cluex* mosquitos, and most were once maintained by animal reservoirs in sylvatic transmission cycles [2]. Many flaviviruses, however, such as dengue virus, yellow fever and Zika virus, are now principally maintained by mosquito-borne transmission with a possible human-to-human transmission through transfusion of infected blood or transplantation of infected tissue [3].

Some flaviviruses can cause globally significant vector-borne diseases with a substantial public health impact such as dengue virus (DENV), Japanese encephalitis virus (JEV), West Nile virus (WNV), Zika virus (ZIKV) and yellow fever virus (YFV) [4]. Other members of the *Flaviviridae* family that have a more regional impact include Murray Valley encephalitis virus (MVEV) in Oceania, St. Louis encephalitis virus (SLEV) in North America, and tick-borne encephalitis virus (TBEV) in Europe [1]. Over the past few decades, many of these flaviviruses have re-emerged for a range of reasons including decreases in mosquito control efforts, rapid changes in climate and vector's demography, dense urbanization, population growth and globalization with increased transportation and trade activities [5]. Examples include the geographic spread of DENV throughout the tropical world; JEV throughout south Asia, Australasia and the Pacific; ZIKV into South and Central America; YFV into the Americas and the invasion of WNV into much of North America [1, 5].

It is estimated that there are over 390 million DENV infections per year, of which 96 million manifests clinically with varying degrees of severity [6] and 3.9 billion people in 128 countries are at risk of infection [7]. Similarly, high incidence rates for symptomatic cases of JEV were reported over the past three decades to reach 2.4 per year per 100,000 population [8]. Epidemic waves of YFV are projected to result in 30,000 to 200,000 clinical cases per year with case-fatality rates ranging from 2 to 15% [9–12]. WNV, first appeared in the northeastern USA in 1999, are spread presently across much of the USA and southern Canada. For example, in 2015, the CDC reported 2,175 cases of WNV, of which 1,616 (74%) were hospitalized and 146 (7%) died [13]. In the developing world, WNV incidence is likely to be underestimated due to political, psychological, and economic barriers to reporting [12, 14].

Although most human flavivirus infections are asymptomatic or have an undifferentiated febrile illness, a small percentage of affected individuals develop acute fever that can progress to severe clinical manifestations such as hemorrhage, vascular leakage and encephalitis [15]. Currently, our knowledge of the host-related factors that influence the pathogenesis of severe disease is inadequate to allow prediction of who will develop severe clinical illness. However, some mechanisms and etiological factors underlying inter-individual variations in response to flavivirus infections have been identified. Interactions of virus-encoded proteins with human innate immune pathways [15]; the effect of host-cell surface molecules in virus binding and entry [16]; the role of viral protein nuclear localization in the host cell response [17]; and the flavivirus replication dynamics within multiple immune systems [18] have all been considered as host-pathogen interaction events that may regulate viral virulence or attenuation and the subsequent disease severity. Over the past few years, however, host-related factors such as preexisting chronic conditions, e.g., cardiovascular diseases, diabetes, obesity, and asthma have received attention as predictors for increased risk of progression to severe flavivirus infection [19–21]. Recent studies have raised the proposition that cardiovascular disease, stroke, diabetes, respiratory diseases and renal disorders may contribute, together with old age, to severe clinical manifestations of dengue [19, 20]. A few studies of WNV [14] and JEV [22] infections, and responses to YFV vaccination [23], have also explored the role of chronic comorbidities in the prognosis of infections. Given the lack of specific medical treatment for flavivirus diseases, effective public health surveillance for vector-borne infections together with continuing vector control efforts will be critical to preventing infection. However, elucidating the impact of comorbidities to the severity of disease when infection occurs will be critical to identifying vulnerable populations, to whom effective interventions protocols and individually-tailored clinical monitoring practices should be particularly targeted.

The objective of this study is to systematically review the existing literature on the prevalence of the most common non-communicable comorbidities related to the cluster of metabolic syndromes-associated diseases, such as diabetes mellitus, heart diseases, hypertension, asthma, stroke and obesity in flavivirus infections and to evaluate the difference of their prevalence in severe vs. non-severe clinical outcomes to infection. Identifying and characterizing associations between comorbidities and severity of flavivirus infections will be significant factor in designing public health measures that aim to prevent the severe outcomes of infection.

## Methods

### Search strategy and selection criteria

**Fig 1:**
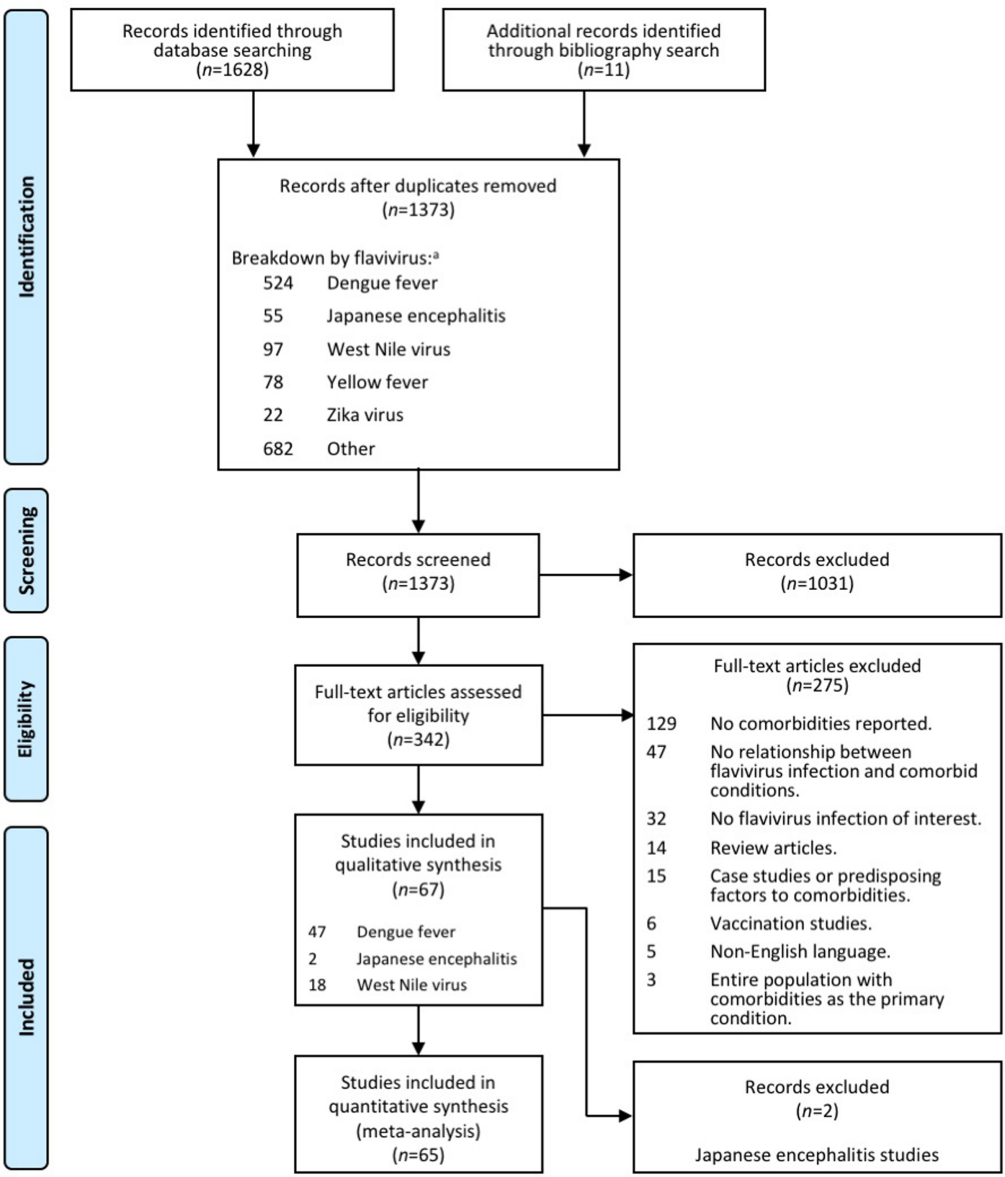
Flowchart of study selection and systematic literature review process. The flow diagram describes the systematic review of literature on the prevalence of comorbidities in flavivirus infections. A total of 65 unique studies were identified (47 studies for dengue fever and 18 for West Nile virus from an initial 1373 examined titles). aSome studies reported on more than one flavivirus disease. Studies drawn from the same population were not included in the meta-analyses.

We carried out a systematic literature search that conforms to the Preferred Reporting Items for Systematic reviews and Meta-Analysis (PRISMA) guidelines [24] (See S1 Table), in PubMed, Ovid MEDLINE(R), Embase and Embase Classic databases from inception to the last week of November 2016 (November 25, 2016). The date for searching database is the same date for the upper limit of the period considered. A grey literature search was also conducted in the American Society of Tropical Medicine and Hygiene and Open Forum Infectious Diseases—Infectious Diseases Society of America for the two most recent years. Using the PICO format (acronym for “population or problem”, intervention or exposure of interest”, “comparison” and “outcome”) [25], the research questions were: "what is the frequency of chronic comorbidities in flavivirus infections?" and “is the severity of flavivirus infection associated with higher prevalence of comorbidities?” (S2 Table). The search related terms (MeSH), inclusion and exclusion criteria and synonyms are shown in Table 1. Non-English language reports were excluded from this study. To identify relevant studies, we used the comprehensive four-step search strategy of PRISMA (Fig 1), *i*.*e*., (*i*) identification, (*ii*) screening, (*iii*) eligibility and (*iv*) inclusion of studies. We evaluated 47 studies in DENV [26–72], 18 in WNV [73–90] and two in JEV [22, 91] for inclusion (see Fig 1). Given the small number of studies in JEV, the two articles were excluded from further quantitative analysis. No studies met the inclusion criteria for YFV and ZIKV infections.

**Table 1:** Keywords for the search related terms and synonyms.

Keyword	Related terms and synonyms
Population	Individuals with a flavivirus infections (both severe and non-severe cases), all age groups.
Flavivirus infection	Dengue fever, Yellow fever, West Nile virus, Zika, Japanese encephalitis.
Comorbidities	Diabetes, hypertension, heart disease (including: cardiovascular disease, coronary artery disease, coronary vascular disease, atrial fibrillation, chronic ischemic heart disease, acute coronary syndrome for duration less than 6 months, cardiac disorder, cardiac heart failure, congestive cardiac failure), stroke, obesity, asthma (does not include chronic obstructive pulmonary disease; COPD).
Proportion/rate	Frequency of at least one of the comorbidities, percent comorbidity, death/fatality, incidence, hospitalization, mortality, mortality rate.
Study design	Retrospective observational, retrospective non-randomized observational, cross-sectional, prospective observational, national surveillance, record-based case-control, matched case–control, age- and sex-matched case control, enhanced surveillance, retrospective cause-of death review, Nested case-control.
Inclusion	All patients to have one or more flaviviruses infections (Dengue, West Nile, Yellow Fever, Zika or Japanese Encephalitis). Studies reporting the frequency of at least one comorbidity (e.g., diabetes, hypertension, heart diseases, stroke, obesity (and/or overweight) or asthma.
Exclusion	Review articles (systematic or narrative), letters, case studies, editorials, vaccination trials, family-based studies, over-counting of the same patient cohort, cases in travelers, animal studies, frequency of comorbidity is not reported, entire study population has chronic disease condition and not flavivirus infection (e.g. people with diabetes where 100% of patients have Dengue), No relationship between chronic disease condition and flavivirus infection mentioned, duplicate studies, non-English literature.

### Inter-reviewer agreement

The titles and abstracts of the identified studies were reviewed independently by two reviewers using Covidence Systematic Review software, (Veritas Health Innovation, Melbourne, Australia. Available at www.covidence.org). Differences and conflicts were resolved by a third reviewer and through discussions for a consensus to be reached. Percentage agreement and Cohen’s Kappa (κ) statistic [92] were calculated and interpreted in accordance with Landis and Koch’s benchmarks for assessing the agreement between reviewers [93] as poor (<0), slight (0.0–0.20), fair (0.21–0.40), moderate (0.41–0.60), substantial (0.61–0.80), and excellent (>0.81).

### Quality score assessment

The methodological quality of each study—from the standpoint of evaluating the prevalence of chronic comorbidities in flavivirus infections—was assessed as part of the data extraction. With some modification, we used the standards tool for evaluating and reporting epidemiologic studies on chronic disease incidence or prevalence, designed to assess population-based prevalence studies [94–96]. To assess the risk of bias, each study was rated against each of the ten following criteria: 1) clearly stating the research objective, 2) distinctly defining the study population, 3) homogeneity of the study subjects (*e*.*g*., age, ethnicity, gender ratio and origin of the studies population), 4) sample size and power justification, 5) assessing the infection prior to identifying the comorbidity, 6) evaluating levels of infectious disease severity and the related frequency of comorbidity, 7) standardizing the infectious disease assessment across all study subjects, 8) standardizing the assessment of comorbidities across all study subjects, 9) assessment of comorbidities blindly of the infectious disease status, and 10) considering the potential confounders with clear and adjusted statistical analyses. Each criterion was rated dichotomously (yes: low risk = 1 point; no: high risk = 0 point). An overall score was calculated by adding all the items rated as low risk. Thus, higher scores indicated lower risk of bias and stronger method quality. Grey literature (mainly conference abstracts) was not assessed in this way as the required information was unavailable.

### Definitions and data extraction and analysis

Data extracted from the selected studies in duplicate by two reviewers and included the first author’s name, publication date, country, dates of recruitment, total sample size (divided to males and females), age estimates (from reported mean, median or the mid-point for age range of the highest subject frequency), procedures for case identification, type of flavivirus infection, severity of infection, prevalence of clinical manifestations (mild symptoms such as fever, headache, muscle pain, rash, and malaise together with severe symptoms as described below) and percentage of comorbidities including diabetes (both type I and type II, if mentioned), hypertension, heart diseases (due to the small sample size of individual conditions, we combined this category from acute coronary syndrome for duration less than 6 months [32 cases; *i*.*e*., 9.3% of all heart disease cases], cardiac diseases [28 cases, 8.1%], cardiac Heart failure and ischemic heart disease [54 cases; 15.7%], cardiovascular diseases [146 cases; 42.4%], congestive cardiac failure [5 cases; 1.5%], heart failure and cardiac disorder [37 cases; 10.8%] and ischemic heart disease [42 cases; 12.2%]), asthma (not including chronic obstructive pulmonary disease), stroke, and obesity and overweight, i.e., BMI >25 kg/m^2^ (Table 1). All DENV and WNV were confirmed cases in the selected studies and infection was identified either by RT-PCR, ELISA, report reviews or from national surveillances. Chronic comorbidities were either self-reported or record-based and did not allow for differentiating between those diagnosed before, after or during the infectious episodes. Weighted average was used to calculate the overall age. Severe cases of DENV were defined as those with any form of the disease that develops major complications such as dengue hemorrhagic fever (DHF, grades I and II), dengue shock syndrome (DSS) (DHF grades III and IV), infections developing organ failures (e.g., acute renal failure and acute respiratory failure), clinically significant bleeding, cases requiring ICU hospitalization and/or fatal cases [26–72]. Severe cases of WNV were defined as those developed WNV neuroinvasive diseases, such as, cases of WN encephalitis (WNE), WN meningitis (WNM), poliomyelitis or acute flaccid paralysis, cases requiring ICU hospitalization, need for rehabilitation, WNV-associated retinopathy (WNVR), chorioretinitis or fatal cases [73–90]. To measure the prevalence rates of comorbidities, we extracted the proportions or percentages reported in the selected studies from the total number of flavivirus cases and from non-severe and severe cases.

Publication bias was assessed by the visual inspection of funnel plot (S1 Fig). Egger’s test [97] was also used to assess publication bias and the tendency for the effects estimated in small sample size studies to differ from those estimated in larger studies. The results of Egger’s test were presented as *t*-value and *P* for publication bias for studies reporting the comorbidity of interest (S4 Table). Significant Egger's test for publication bias was based on *p*<0.1. The primary outcome measure was to evaluate the overall prevalence of comorbidities in the flavivirus infection and the estimates stratified by severity. Meta-analyses were carried out to assess the pooled prevalence (and 95% CI) of clinical symptoms and the proportions of each comorbidity in the total, severe and non-severe cases of each infection.

Meta-analysis tests were conducted using Comprehensive Meta-Analysis software, CMA version 3.9 (Englewood, NJ, USA) [98]. Variances of raw proportions or percentages were pooled based on a binary random-effects model [99], given the population heterogeneity and assuming the relationships between comorbidities and flavivirus infections vary across populations. Forest plots were used to illustrate the prevalence of comorbidities in flavivirus infections from the selected studies prior to stratifying the proportions by severity. Comparisons of proportions for the prevalence of clinical symptoms between diseases and the pooled prevalence estimates of comorbidities in relation to severity were carried out using the Chi-squared test as previously recommended [100, 101]. ORs adjusted for age (and 95% CI) for severe infection outcome in patients with comorbidities were calculated using the Cochran-Mantel-Haenszel test as previously described [102–104]. All statistical tests were two-sided and conducted using SPSS Statistics, Version 21.0 (SPSS Inc., Chicago).

Assessing the heterogeneity among the selected studies was carried out using the *Q* test [105] that informs about the presence versus the absence of heterogeneity. The *Q* test, however, does not report the extent of heterogeneity and has inadequate power to detect heterogeneity among the small number of studies identified for some comorbidities. Therefore, we calculated the *I*^*2*^ index to complement the *Q* test to describe the degree of between-study heterogeneity [106]. *I*^*2*^ index values were categorized as low (0–30%), moderate (30–60%), substantial (60–90%), and considerable (>90%) as recommended [107]. We also quantified the true heterogeneity by estimating the within-study variance in the random-effects model (τ^2^), as previously described [108].

### Data availability

All data generated or analyzed during this study are included in this published article (and its supporting information files).

## Results

The original search resulted in 1,628 articles selected for title review as they satisfied our selection criteria (S3 Table). Additional 11 articles were identified through bibliography search from previously identified systematic reviews (Fig 1). After de-duplication, a total of 1,373 original titles were selected for abstract review. Abstract review resulted in the exclusion of 1,031 reports. Full text assessment was conducted on the remaining 342 articles by at least two reviewers and resulted in the selection of 47 studies in DENV [26–72], 18 in WNV [73–90] and two in JEV [22, 91] (those were further excluded) for inclusion (see Fig 1 for exclusion criteria and breakdown). The agreement on the inclusion between two reviewers was 86.5% with weighted κ = 0.62 (95% CI: 0.52–0.72). This substantial agreement (0.61–0.80) may relate to the extent of clarity in the assessed abstracts when reporting the rates or the presence of comorbidities at the initial stage of the selection process. No studies were identified, however, for YFV and ZIKV infections. After excluding the 2 studies on JEV, the quality of each of the remaining 65 studies, involving 61 sets of patients, was assessed. With a maximum quality score of 10, a good score (≥7 points) was achieved in 35% of the studies (16 studies on DENV and 7 on WNV); 30% of the studies were scored as being of fair quality (5–7 points including13 studies on DENV and 6 on WNV) and 20% of the studies were scored as low quality (≤5 points including 11 studies on DENV and 14 on WNV) (Table 2). No score was assessed for the remaining 9 reports from the grey literature (abstracts; 7 on DENV and 2 on WNV). More than 70% of the reports (48 studies) were retrospective in nature with the rest being age- and sex-matched or nested case-control studies, surveillance reports, or prospective studies. In these studies, there was a wide variation in the sample size ranging from five [81] to 6,070 [46] patients.

**Table 2:** Characteristics of studies included in meta-analysis for prevalence of chronic comorbidities in flavivirus infections.

Infection	Study ID	Dates of recruiting (mm.yy or yyyy)	Study design	Case identification procedure^1^	Country	Number of study subjects			Age estimate (years)	Research quality^6^
						Total	Males	Females		
**Dengue Fever**										
	Chen *et al*., 2016 [26]	07.15–11.15	Retrospective	Laboratory confirmed	Taiwan	143	70	73	69.7	Good
	Lee *et al*., 2016 [27]	01.05–12.08	Retrospective non-randomized observational	RT-PCR	Singapore	788	575	213	40	Good
	Mercado *et al*., 2016 [28]	09.14–10.15	Retrospective	Report review	Colombia	7	3	4	-	Low
	Mirza *et al*., 2016 [29]	10.11–11.11	Retrospective	Admission record	Pakistan	563	215	348	48.5	Good
	Rosenberger *et al*., 2016 [30]	08.06–05.07	Prospective	Case report review	Multiple^**2**^	1734	924	810	7	Fair
	Tedesco *et al*., 2016 [31]	-	Prospective	-	Ecuador	72	-	-	-	NA
	Wei *et al*., 2016 [32]	01.14–12.14	National surveillance	RT-PCR	Taiwan	136	66	70	71	Fair
	Wong *et al*., 2016 [33]	01.05–12.08	Retrospective	RT-PCR	Singapore	4383	2924	1459	34.1	Good
	Woon *et al*., 2016 [34]	01.13–12.14	Retrospective	Report review	Malaysia	320	155	165	40.7	Low
	Mallhi *et al*., 2016 [35] Mallhi *et al*., 2015a [36] Mallhi *et al*., 2015b [37]	01.08–12.13	Retrospective (duplicate)^**5**^	Report review	Malaysia	667	378	289	30.7	Fair—Good
	Aamir *et al*., 2015 [38]	10.02–11.02	Retrospective	-	Pakistan	100	73	27	34.5	Low
	Chen *et al*., 2015 [39] Lee *et al*., 2006 [70]	06.02–12.02 01.02–12.02	Retrospective (duplicate)^**5**^	RT-PCR and record review	Taiwan	644	295 296	349 348	47.6	Fair—Good
	Chen *et al*., 2015 [39]	06.02–12.02	Retrospective	RT-PCR	Taiwan	644	295	349	47.6	Fair
	Huang *et al*., 2015 [40]	06.05–07.05	Retrospective	Report review	Taiwan	1076	498	578	53	Fair
	Kutsuna *et al*., 2015 [41]	08.14–09.14	Retrospective	Report review	Japan	19	10	9	33	Low
	Fernandes-Charpiot *et al*., 2014 [42]	01.10–12.13	Retrospective	Report review	Brazil	11	7	4	48	NA
	Fujimoto and Koifman, 2014 [43]	01.07–06.11	Retrospective	Serology	Brazil	193	110	83	38.2	Low
	Iqtadar *et al*., 2014 [44]	-	Retrospective	Report review	Pakistan	150	51	99	-	NA
	Karunakaran *et al*., 2014 [45]	06.05–06.08	Retrospective case-control	RT-PCR	India	50	29	21	-	Low
	Ng *et al*., 2014 [46]	2005–2008	Retrospective	RT-PCR and serology	Singapore	6070	3927	2143	-	NA
	Pang *et al*., 2014 [47]	01.04–12.08	Retrospective Matched case–control	Report review	Singapore	135	88	47	36	Good
	Saqib *et al*., 2014 [48]	06.11–11.11	Retrospective	Report review	Pakistan	556	390	166	36	Fair
	Lee *et al*., 2013 [49]	12.99–01.00	Prospective case-control	ELISA	Taiwan	193	91	103	-	Fair
	Mahmood *et al*., 2013 [50]	09.11–12.11	Age- and sex-matched case control	Report review	Pakistan	132	71	61	49.5	Low
	Mohamed *et al*., 2013 [51]	08.09–12.09	Retrospective	RT-PCR	Yemen	100	54	46	-	Fair
	Pang *et al*., 2013 [52]	2004–2007	Retrospective case-control	Report review	Singapore	135	-	-	-	NA
	Thein *et al*., 2013 [53]	01.04–12.08	Hospital-based retrospective	RT-PCR or NS1 antigen	Singapore	108	59	49	38.3	Good
	Tomashek *et al*., 2013 [54]	2010–2012	National surveillance	-	Puerto Rico	56	22	34	45	NA
	Assir *et al*., 2012 [55]	08.11–11.11	Retrospective—hospital record	Report review	Pakistan	60	41	19	44	NA
	Chamnanchanunt *et al*., 2012 [56]	06.05–07.05	Retrospective	Report review	Thailand	277	165	112	23.1	Good
	Pang *et al*., 2012 [57]	06.07–06.08	Retrospective case-control	Serology	Singapore	2285	1536	749	36.6	Good
	Low *et al*., 2011 [58]	04.05–12.05	Prospective	ELISA	Singapore	250	152	98	39	Fair
	Figueiredo *et al*., 2010 [59]	06.02–6.05	Matched case-control	Report review	Multiple^**3**^	1345	609	736	-	Good
	Laoprasopwattana *et al*., 2010 [60]	01.89–12.07	Retrospective	Report review	Thailand	75	37	38	8.9	Low
	Lye *et al*., 2010 [61]	06.04	Retrospective	Serology	Singapore	1971	1256	715	33.1	Low
	Thomas *et al*., 2010 [62]	01.05–12.08	Prospective observational	RT-PCR	Martinique	560	263	297	37	Fair
	Lee *et al*., 2009 [63] Lee *et al*., 2008 [66]	06.02–12.02	Retrospective (duplicate)^**5**^	RT-PCR, ELISA, serology	Taiwan	304 307	137 139	167 168	53.4	Good
	Kuo *et al*., 2008 [64]	01.02–01.03	Retrospective	RT-PCR, ELISA	Taiwan	519	265	254	48	Good
	Lahiri *et al*., 2008 [65]	12.04–11.05	Retrospective	Report review	Singapore	9	6	3	56.1	Fair
	Liu *et al*., 2008 [67]	06.02	Retrospective	RT-PCR, ELISA	Taiwan	155	77	78	51.6	Fair
	Passos *et al*., 2008 [68]	12.01–04.02	Retrospective	Serology	Brazil	453	195	258	35.7	Good
	Wang *et al*., 2007 [69]	06.02–12.02	Retrospective	RT-PCR, ELISA	Taiwan	606	268	338	50.7	Fair
	Kalayanarooj and Nimmannitya, 2005 [71]	06.95–06.99	Retrospective	ELISA	Thailand	4532	-	-	7.9	Low
	Cunha *et al*., 1999 [72]	01.97–11.97	Observational	ELISA	Brazil	24	-	-	-	Low
	**Total/Weighted average±S.D.**					**31,969**	**16,095**	**11,112**	**30.8±14.5**	
**West Nile virus**										
	Hasbun *et al*., 2016 [73]	06.02–07.12	Prospective	Surveillance	USA	111	60	51	59.2	Good
	Weatherhead *et al*., 2015 [74]	06.05–06.06	Longitudinal	ELISA	USA	60	38	22	60.5	Good
	Pem-Novosel *et al*., 2014 [75]	09.12	Retrospective	Serology	Croatia	7	3	4	62.0	Low
	Racsa *et al*., 2014 [76]	05.12–09.12	Retrospective	Serology	USA	57	30	27	52.0	Fair
	Vrioni *et al*., 2014 [77]	08.11–10.11	Retrospective	RT-PCR, ELISA	Greece	31	22	9	63.3	Fair
	Hoffman *et al*., 2013 [78]	01.02–12.09	Retrospective	-	USA	48	29	19	67.8	Fair
	Mora *et al*., 2013 [79]	05.12–12.12	Retrospective	-	USA	19	12	7	**-**	NA
	Popovic *et al*., 2013 [80]	08.12–10.12	Cross-sectional	Report review	Serbia	58	40	18	61.0	Good
	Sakagianni *et al*., 2013 [81]	08.11–0812	Retrospective	Report review	Greece	5	4	1	74.0	NA
	Lindsey *et al*., 2012 [82]	06.08–06.10	Enhanced retrospective surveillance	Report review	USA	1090	615	475	**-**	Good
	Danis *et al*., 2011 [83]	06.10–10.10	Observational	ELISA	Greece	33	23	10	72.0	Good
	Sejvar *et al*., 2011 [84]	06.02–06.06	Retrospective cause-of death review	Report review	USA	23	14	9	78.0	Low
	Cook *et al*., 2010 [85]	06.06–06.08	Retrospective cross-sectional	Serology	USA	265	106	159	51.7	Good
	Papa *et al*., 2010 [86]	07.10–08.10	Observational	Surveillance	Multiple^**4**^	81	45	36	70.0	Low
	Murray *et al*., 2009 [87]	01.02–12.04	Observational case-control	Report review	USA	113	80	33	64.0	Fair
	Jean *et al*., 2007 [88]	05.05–11.05	Retrospective	Surveillance	USA	839	458	381	**-**	Fair
	Khairallah *et al*., 2007 [89]	08.03–11.03	Prospective	ELISA	Tunisia	38	22	16	60.8	Fair
	Murray *et al*., 2006 [90]	06.02–11.04	Retrospective nested case-control	ELISA	USA	172	115	57	54.0	Good
	**Total/Weighted average±S.D.**^**7**^					**3,050**	**1,716**	**1,334**	**59.1±14.9**	

^**1**^In the report review procedure; all cases of dengue fever and West Nile virus are confirmed.

^**2**^Thailand, Philippines, Vietnam, Malaysia, Nicaragua, Venezuela, Brazil

^**3**^Salvador and Brazil

^**4**^Macedonia and Greece

^**5**^These studies are likely to be drawn from the same population. As this may result in repeated case counting, if duplicate studies have different number of cases, the study with the smaller sample size was not included into the total number of subjects or the analysis of the weighted average age ± S.D.

^**6**^Research quality is assessed from the perspective of evaluating the prevalence of chronic comorbidities in flavivirus infections (see Methods section). NA, not assessed (grey literature).

^**7**^Age is significantly different between the two diseases (*p*<0.0001, *t*-test)

Same cohort was likely to be reported for DENV in two occasions, i.e., by Chen et al., 2015 [39] and Lee et al., 2006 [70] and also by Lee et al., 2009 [63] and Lee et al., 2008 [88]. To avoid repeated counting of the cases from the same study cohort, we only included the study with the larger sample size both in evaluating the total number of subjects and in the analysis of average age of the studied cases. Furthermore, one study [30] reported that out of the 1,734 studied DENV cases, about 10% were classified as highly suggestive whereas the remaining 90% were confirmed by the diagnostic algorithm. This report did not separate or identify this small number of cases (~170 cases) that represents only 0.53% of the total number evaluated here and were included into our analysis. Moreover, one study [28] included fatal cases of DENV and chikungunya virus coinfection. Although these cases may represent an unusual clinical population, they were included here for our report to be inclusive as the number of subjects was extremely small (*n* = 7) and they only had 3 cases of hypertension (representing <0.08% of the entire hypertensive cases). To examine if the study estimates are related to the size of the study, publication bias was assessed by visual inspection of funnel plots (S1 Fig) and by Egger's test (S4 Table). Funnel plot inspection demonstrated a seemingly non-symmetrical distribution of the effect size on either side of the pooled estimate, suggesting some evidence of publication bias. Results of Egger’s test, however, for most of the associations between DENV or WNV and the comorbidities showed *p*>0.1 (except for the prevalence of hypertension in DENV and heart diseases in WNV, *p* = 0.03), given the assumption for evidence of small-study effects is based on *p*<0.1 as previously reported [109].

The studies selected for DENV were geographically diverse and included 18 countries. The reports were from Southeast Asia, South America, Eastern Mediterranean, and Western Pacific Regions. Most of the WNV reports were, however, from the United States with few studies from Europe. Overall, the prevalence rates of comorbidities were reported in 34,949 individuals (31,969 for DENV and 3,050 for WNV). The majority of the studies provided gender breakdown, with a combined male:female ratio ranging from 1.3 for WNV to 1.4 for DENV. The overall weighted average age (±S.D.) of DENV cases (30.8±14.5 years) was significantly younger (*p*<0.0001, *t*-test) than those with WNV (59.1±14.9 years). Comparison of pooled estimates of clinical symptoms in DENV and WNV at the time of presentation showed significantly higher frequencies of fever, headache, and rash in DENV than WNV and lower prevalence of malaise (Table 3). As expected, febrile illness was the most frequent clinical symptom in DENV (96.9% of the cases, 95% CI: 92.5–98.8%) and in WNV (77.1%, 95% CI: 19.8–97.9%). Except for headache in DENV, a low to moderate *I*^*2*^ index (0–60%) was obtained for the combined prevalence estimates of all DENV and WNV clinical symptoms, indicating low degree of heterogeneity among studies. In the selected set of studies, the most prevalent severe DENV forms were the DHF/DSS (21.7%, 95% CI: 14.4–29.1%) followed by undergoing for platelet transfusion (21.1%, 95% CI: 1.5–40.8%) (Table 4). In WNV, however, meningitis and encephalitis were present in >35% of the severe cases, representing the highest prevalent severe condition.

**Table 3:** Meta-analysis for the prevalence of clinical symptoms in flavivirus infections in the selected studies.

Infection		Clinical symptoms^1^				
		Fever	Headache	Muscle Pain	Rash	Malaise
**Dengue fever**						
	Prevalence (%)	96.9 (28)	50.8 (22)	48.0 (16)	30.8 (24)	33.4 (4)
	95% CI (%)	92.5–98.8	42.8–58.7	34.2–62.0	26.3–35.6	12.0–64.8
	*n*	22,053	14,158	9,656	12,805	1,957
	*Q*	15.0	68.6	19.0	56.0	3.6
	*I*^*2*^	0.0	69.4	26.2	60.7	16.4
	*τ*^*2*^	136.5	12.0	17.4	5.4	6.9
**West Nile Virus**						
	Prevalence (%)	77.1 (5)	38.9 (4)	49.3 (2)	13.9 (2)	51.1 (2)
	95% CI (%)	19.8–97.9	17.8–65.2	5.6–94.1	8.7–21.5	20.0–81.4
	*n*	423	399	1,104	115	323
	*Q*	3.9	1.9	1.0	0.001	1.0
	*I*^*2*^	0.0	0.0	0.0	0.0	0.0
	*τ*^*2*^	40.2	4.5	8.1	0.0	2.0

^1^Number in parenthesis represents the number of studies from which prevalence was extracted.

**Table 4:** Meta-analysis for the frequency of clinical features in severe flavivirus infections in the selected studies.

Infection	Clinical Feature	Number of Studies	*n*	Prevalence (%)	95% CI (%)	Analysis of heterogeneity		
						*Q*	*I*^*2*^	*τ*^*2*^
Dengue fever	Organ Involvement	6	1056	24.3	-1.6–50.2	1079	99.5	0.10
	Dengue hemorrhagic fever/Dengue shock syndrome	18	5220	21.7	14.4–29.1	1649	98.9	0.02
	Platelet transfusion	4	3150	21.1	1.5–40.8	1046	99.7	0.04
	Severe plasma leakage	2	270	17.9	-11.9–47.6	8	87.7	0.04
	Severe bleeding	8	5582	14.1	9.3–18.9	201	96.5	0.03
	ICU	6	4425	7.6	3.1–12.0	218	97.7	0.03
	Death	11	5288	3.0	1.7–4.3	130	92.3	0.00
West Nile Virus	Meningitis/meningoencephalitis	5	146	37.2	6.7–67.7	119	96.6	0.12
	Encephalitis	5	270	35.4	15.4–55.5	262	98.5	0.05
	Acute respiratory failure	2	77	29.8	11.1–48.4	3	59.2	0.01
	Flaccid paralysis	4	109	13.4	2.3–24.5	9	65.1	0.01
	Nuroinvassive conditions	3	3282	7.1	1.2–12.9	43	95.4	0.02
	ICU	2	3010	2.2	-3.6–8.1	3	68.1	0.00
	Death	5	3263	8.0	3.8–12.2	13	69.9	0.01

**Fig 2:**
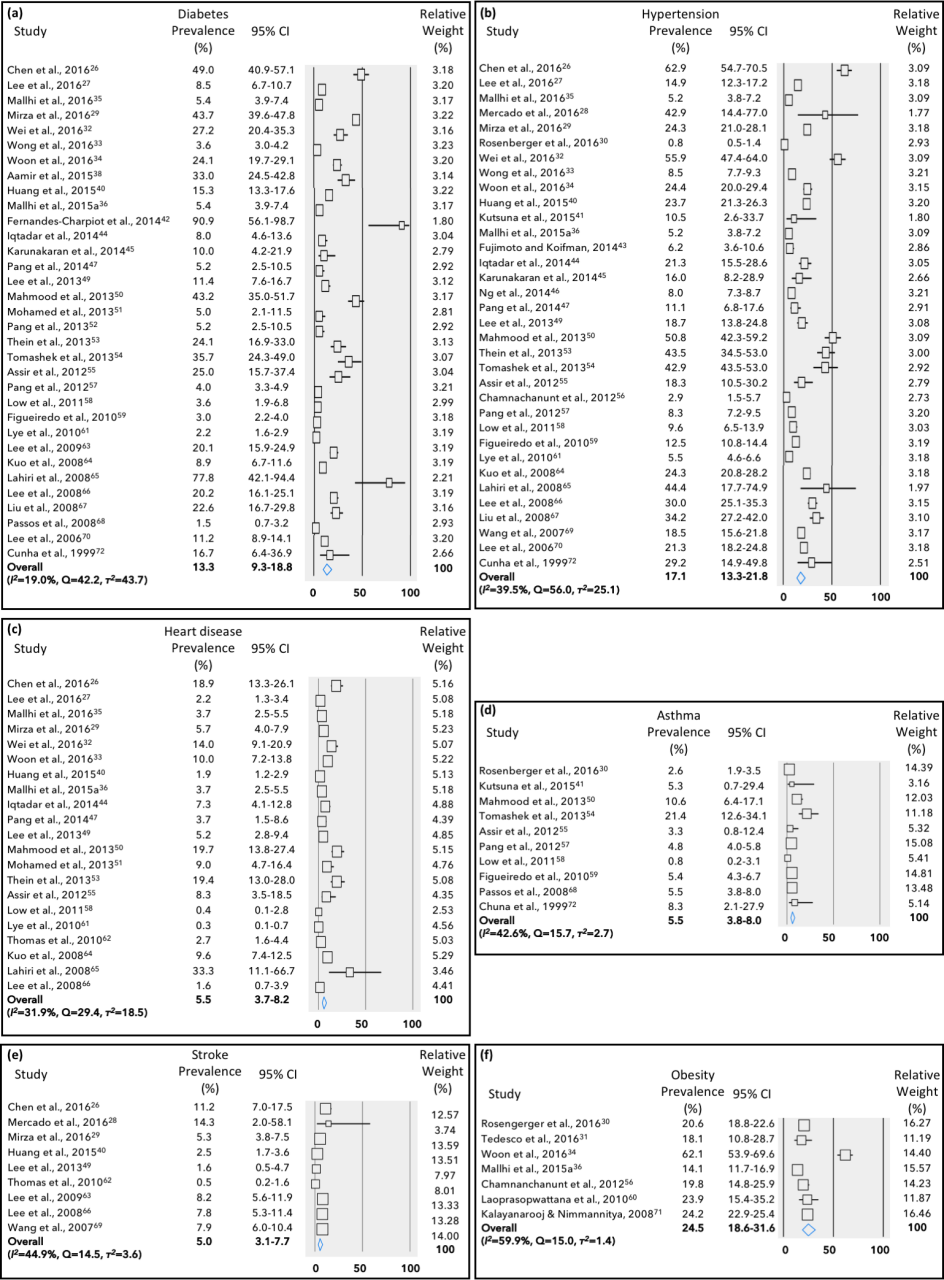
Meta-analysis for the proportion of comorbidities in dengue fever cases. Weights are calculated from binary random-effects model analysis. Values represent proportion of diabetes (a), hypertension (b), heart diseases (c), asthma (d), stroke (e) and obesity/overweight (f) in dengue fever patients and 95% CI. Heterogeneity analysis was carried out using *Q* test, the among studies variation (*I*^*2*^ index) and within-study variance in the random-effects model (τ^2^).

**Fig 3:**
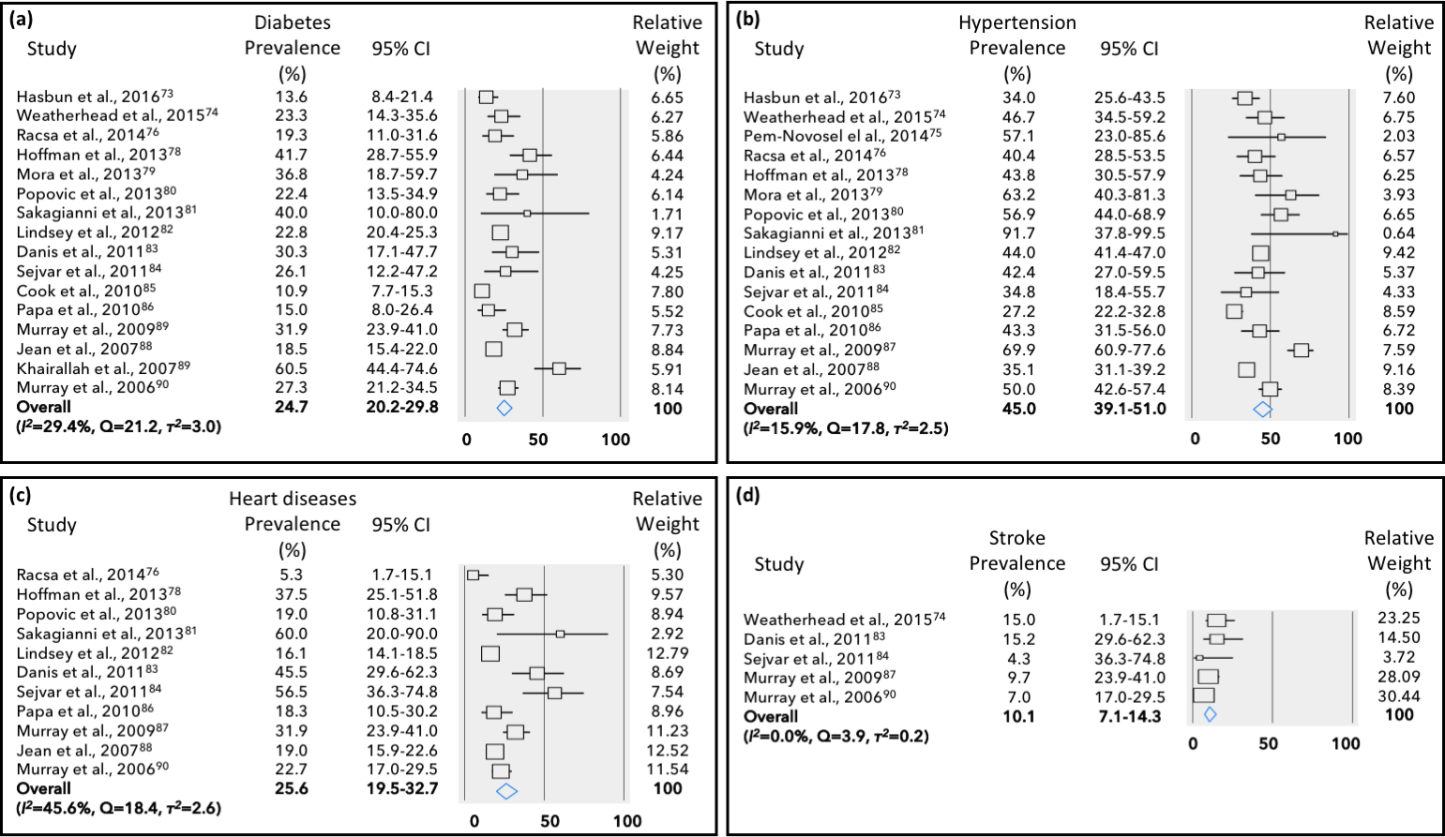
Meta-analysis for the proportion of comorbidities in West Nile virus cases. Weights are calculated from binary random-effects model analysis. Values represent proportion of diabetes (a), hypertension (b), heart diseases (c) and stroke (d) in West Nile virus patients and 95% CI. Heterogeneity analysis was carried out using *Q* test, the among studies variation (*I*^*2*^ index) and within-study variance in the random-effects model (τ^2^).

Obesity/overweight was the most prevalent comorbidity in DENV patients (24.5%, 95% CI: 18.6–31.6%), followed by hypertension (17.1%, 95% CI: 13.3–21.8%) and diabetes (13.3%, 95% CI: 9.3–18.8%) (Fig 2). Heart disease, asthma and stroke were present in about 5.0% of the DENV cases. On the other hand, in WNV cases, hypertension was the most frequent comorbidity (45.0%, 95% CI: 39.1–51.0%), followed by heart diseases (~25%), diabetes (~25%) and stroke (10.1%, 95% CI: 7.1–14.3%) (Fig 3). No study in WNV has reported the incidence of obesity or asthma. Overall, there was low (*I*^*2*^ = 0–40%) to moderate (*I*^*2*^ = 30-60%) heterogeneity among the identified studies.

When cases of DENV and WNV were stratified by severity (Table 5), there was 3- to 4-fold higher prevalence (*p*<0.0001) of diabetes and heart diseases in severe DENV and WNV cases, respectively compared to their rates in the non-severe disease. Hypertension, however, was about 2-fold more prevalent in severe cases of both flavivirus infections (*p*<0.0001) than non-severe cases. Asthma and obesity were only examined in DENV as none of the selected studies reported their prevalence in WNV. Asthma and obesity were ~1.5-fold more frequent (*p*<0.001) in patients with severe DENV than in non-severe cases. The frequency of stroke was assessed only in one study in non-severe DENV [69] and WNV [74], which did not permit comparison between severe and non-severe cases. Based on the differences in prevalence of comorbidities between severe and non-severe cases of DENV and WNV, we estimated the age-adjusted OR of severe infection outcome in patients with chronic comorbidities to be 3.41 (95%CI: 2.54–4.59) in DENV patients with obesity/overweight, 2.76 (95%CI: 2.54–299) in patients with diabetes, 2.4 in those with heart diseases (95% CI: 1.65–1.70) and 1.61 (95% CI: 1.52–1.70) in patients with hypertension. The OR of severe WNV was 4.21 in patients with diabetes (95% CI: 2.22–7.92) and 2.72 in those with hypertension (95% CI: 1.78–4.14) (Table 5).

**Table 5:** Pooled prevalence estimates of comorbidities in severe and non-severe cases of flavivirus infections dengue fever and West Nile virus.

Infection	Comorbidity	Severe infection						Non-Severe infection						*p*^1^	OR_adj_ (95% CI)^2^
		Number of Subjects	Number of studies	Prevalence (%) (95% CI)	Analysis of heterogeneity			Number of subjects	Number of studies	Prevalence (%) 95% CI	Analysis of heterogeneity				
					*I*^*2*^ (%)	Q	*τ*^*2*^				*I*^*2*^ (%)	Q	*τ*^*2*^		
**Dengue fever**	Diabetes	3236	22	22.6 (16.3–30.5)	0.99	21.2	17.8	9067	13	5.8 (2.9–11.1)	0.0	8.9	19.7	<0.0001	2.76 (2.54–2.99)
	Hypertension	3497	24	28.0 (21.1–36.1)	15.3	27.2	17.9	9055	12	12.3 (8.7–17.1)	52.2	23.0	4.7	<0.0001	1.61 (1.52–1.70)
	Heart diseases	991	10	15.8 (11.4–21.5)	17.4	10.9	2.4	819	4	4.0 (1.5–10.0)	0.0	2.8	2.6	<0.0001	2.14 (1.65–2.77)
	Asthma	1260	6	7.8 (4.3–14.0)	0.0	4.2	2.9	2642	2	5.1 (4.4–6.1)	0.0	0.1	0.0	0.0009	1.12 (0.74–1.54)
	Stroke	465	4	10.9 (7.1–16.2)	7.6	3.2	0.3	595	1	7.6 (5.7–10.0)	-	-	-		1.31 (0.85–2.01)
	Obesity/overweight	4026	5	28.3 (15.8–45.5)	0.0	3.9	3.4	1584	3	18.3 (12.4–26.2)	0.0	1.3	0.4	<0.0001	3.41 (2.54–4.59)
**West Nile virus**	Diabetes	1456	15	28.9 (22.8–35.9)	24.8	18.6	3.9	878	7	9.6 (7.8–11.7)	0.0	6.0	0.0	<0.0001	4.21 (2.22–7.93)
	Hypertension	1431	14	43.9 (35.2–53.0)	24.1	18.4	5.6	869	6	27.4 (24.2–31.0)	0.4	5.1	0.0	<0.0001	2.72 (1.78–4.14)
	Heart diseases	1329	11	24.1 (17.2–32.6)	42.1	17.3	3.9	778	4	8.1 (3.2–19.3)	0.0	2.5	2.4	<0.0001	6.67 (0.91–50.3)
	Stroke	277	4	11.1 (5.9–18.8)	0.0	3.0	1.0	11	1	4.2 (0.3–42.5)	-	-	-		5.92 (0.76–45.9)

^**1**^Comparison of prevalence in relation to severity, using the Chi-squared test.^100,101^ Only significant values are shown.

^**2**^Odds ratios are adjusted for age using Cochran Mantel-Haenszel test.

## Discussion

The present study evaluates the frequency of comorbidities in DENV and WNV as examples to flavivirus infections that represent a major global health problem. Datasets were few or unavailable for JEV, ZIKV and YFV. Only two studies were found to satisfy our selection criteria for JEV [22, 91] and most of the reports on ZIKV were related to co-existing infectious diseases such as chikungunya or HIV [110–112] or to gestational diabetes in a single case [113]. Studies in YFV, however, primarily evaluated the safety and effectiveness of vaccination (17D-YF) in people with pre-existing chronic illnesses [114–116]. A few systematic reviews on the frequency of comorbidities in flavivirus infection exist and none have used meta-analysis to synthesize global prevalence estimates. We observed a large difference in the volume of literature and total number of patients evaluated for each of the examined condition where 47 studies were identified for DENV [26–72] with 31,969 subjects and 18 for WNV [73–90] with 3,050 patients. Almost 40% of the selected studies were assessed to have good quality score for evaluating and reporting chronic disease incidence or prevalence [94–96]. However, chronic diseases were not clearly defined in the vast majority of the studies as they were either self-reported or record-based and did not allow for differentiating between those diagnosed before, after or during the infectious episodes.

Very few of the selected reports provided sample size justification and all had infectious disease status assessed prior to the chronic disease scoring. The development of chronic diseases following infection was not reported in any of the selected studies. Furthermore, none of the selected studies reported either DENV or WNV as a coinfection for one another. Moreover, a few number of studies provided information on the status of more than one chronic disease comorbidity in the same patient. Additionally, assessment of the chronic diseases was not blindly evaluated with respect to the infectious diseases status. These factors meant that 60% of the selected studies had good-to-fair quality scores. This made us choose to include studies that were assessed to be of "fair" or "low" quality in our meta-analysis to attain more comprehensive estimates with larger sample size and to avoid the bias when excluding studies with small or no effect. The geographical diversity of DENV reports compared to WNV studies likely reflects the fact that dengue now is the most common flavivirus infection globally with transmission occurring in at least 128 countries and almost 4 billion people around the world [7]. WNV, on the other hand, despite being an important cause of human disease worldwide with continual increase in transmission over the past 77 years [5, 14], is spreading in a notably lesser rate than DENV [5]. Only one study of the 18 selected reports on WNV were from regions other than Europe or the USA [89]. Given the repeated WNV outbreaks between 1996 and 2015 in Israel and Europe and its movement through North America over the last decade that resulted in sustained presence in the community [5, 13,14], it was predictable to identify a limited set of WNV studies from regions other than those primarily affected.

Most clinically apparent acute flavivirus infections progress from a classic presentation of mild fever, headache, muscle pain, rash, and malaise to a more disease-specific syndrome of acute febrile illness to further severe states of hemorrhagic fever and encephalitis. Pooled prevalence analysis of these clinical symptoms indicated their comparatively lower frequencies in WNV than in DENV. It is known that WNV infection is commonly asymptomatic, with ~20% of infected persons having clinically apparent disease [117–119]. Symptomatic patients mostly present with fever, headache and malaise and less commonly with myalgia, rash, neck pain, and arthralgia [76]. On the other hand, patients infected with DENV are asymptomatic in the majority of cases but a small proportion may develop an array of clinical symptoms ranging from mild flu-like syndrome, such as fever, skin rash, headache, myalgia, and arthralgia, to severe forms of the disease. Only rash was 2-fold more frequent in DENV than WNV cases. According to the 2009 WHO classification system [120], DENV patients can be clinically classified as with 'probable dengue’, ‘dengue with warning signs’ or ‘severe dengue'. This deviates from the 1997 WHO classification system [121], categorizing patients as with DF, DHF or DSS. Over 90% of the DENV patients in the selected set of studies presented with DF. The progression of DF to severe clinical manifestations is mostly unpredictable. Case fatality rate may exceed 20% if timely and appropriate differential therapy is not introduced to reduce the impact of disease complications [120].

Recent estimates for the global burden of dengue suggest the epidemics mainly affect children and young adults between the ages of 5 and 30 years old in terms of death, years lived with disability (YLDs), years of life lost (YLLs) and disability-adjusted life-years (DALYs) [122]. However, an age shift to more adult cases with severe DENV (and more comorbidities) is presently demonstrated in several reports from around the world (see below) [120, 123, 124]. In addition to this age-shift in severe DENV cases, the incursion of DENV into new world regions may have influenced the age-related increased incidence of the disease. Advanced age, on the other hand, has long been known as a frequent risk factors for severe disease outcome of WNV [14]. Average annual incidence of severe WNV cases reported to CDC between 1999–2015 for cases over 70 years old was ~1.3 per 100,000 population per year compared to only ~0.5 for patients <10–39 years old [125, 126].

Introduction of the competent vectors into natural environments and urban areas, together with the changing societal factors (migration, industrialization, trade, urbanization and population growth), may all have facilitated the geographical expansion of flavivirus diseases into different regions of the world. Examples include the expansion of DENV from the Caribbean islands to Brazil and from the Pacific islands to other regions in south Asia [127, 128] and WNV from Africa and the Middle East to Europe and North America [5]. Many of the regions where these vector-borne diseases spread are also experiencing an epidemiological shift from communicable diseases to non-communicable disorders as the primary causes of the morbidity and mortality [129]. With the ageing population, non-communicable diseases now account for nearly half of the disease burden in low- and middle-income countries [130]. Therefore, flavivirus diseases, particularly DENV, may have been shown now to affect older adults, an age group with inherently more comorbidities [130, 131].

The present study demonstrates that hypertension and diabetes are the most prevalent comorbidity in both DENV and WNV with obesity/overweight and heart diseases being present, respectively, in about 20% of the DENV and WNV cases. Although the prevalence of diabetes, hypertension and heart diseases varied widely among the DENV selected studies (ranging from 60- to 100-fold), the vast majority of the reports showed values clustering around the pooled estimated averages for each comorbidity, as evidenced by the low *I*^*2*^ index values. Obesity, stroke and asthma proportions varied only by 5- to 30-fold among the studies with moderate *I*^*2*^ index values. However, in WNV studies, the among-studies prevalence of comorbidities varied by 3- to 12-fold for diabetes, hypertension, heart diseases and stroke with low to moderate *I*^*2*^ indexes. These variations in the prevalence of comorbidities in WNV cases may relate to the higher average patients' age (compared to DENV) since older age is a risk factor for a number of non-communicable diseases, *e*.*g*., diabetes and heart conditions [132–136]. On the other hand, the wider variation in prevalence of comorbidities in DENV than WNV can be linked to the different patterns of geographical distribution between the two diseases.

Diabetes, hypertension and heart diseases were, respectively, 2- to 4-fold significantly more prevalent in severe DENV and WNV than in non-severe cases. This observation is supported by a number of case-control studies implicating comorbidities in severe outcome of flavivirus infections. Over 2-fold higher frequency of diabetes were found in severe DENV cases than in cases with DF [45, 47, 57, 59]. Furthermore, severe clinical presentation of DENV was likely to develop in patients with diabetes than in non-diabetic subjects with the odds ratio (OR) ranging from 1.26 (95% CI 0.8–2.0) [50] to 26 (95% CI 2.5–273.7) [45]. This is line with the OR of about 2 to 4 for severe DENV and WNV observed here among patients with diabetes or heart diseases. Diabetes risk factors such as glucose intolerance [137] and hyperlipidemia [53] were prevalent in 54% and 17% of severe DENV cases, respectively and more frequent in elder patients [61, 66]. Similarly, diabetes (19–42%), hyperlipidemia (17–19%) and coronary artery disease (5–17%) were prevalent in WNV cases than controls [76, 78] with OR of neuroinvasive outcome varying between 2.4 (95% CI 0.57–10.4) and 4.5 (95% CI 0.88–23.1) [76]. Diabetes was 23% more frequent in cases with WNE and WNM than in WNV fever [74]. Developing encephalitis was more likely to occur in WNV cases who also had diabetes (OR = 2.0; 95% CI: 1.1–3.7) or cardiovascular diseases (OR = 28.3; 95% CI: 5.9–134.9) [87]. Furthermore, hypertension was shown to be present in 50% of the severe or fatal DENV cases [1], the manifestation of acute respiratory failure [6], and in higher proportion of elder patients [1, 3] with OR varying from 1.6 (95% CI 1.1–2.1) [4] to 44.3 (95% CI 6.2–315.5) [5]. In contrast, some studies did not show such a relationship in severe DENV [8, 9, 138]. In WNV, patients with hypertension had higher odds of developing neuroinvasive outcome (OR 1.88, 95% CI 0.63–5.58) [78] and encephalitis (OR = 5.1, 95% CI 2.5–10.4) [87] than non-hypertensive cases. High prevalence of hypertension (46%) was also noted in patients with WNE, WNM and fatal outcome compared to those in WNV fever [74]. In line with these findings, the results of the present study strongly suggest that diabetes, heart diseases and hypertension are more prevalent in severe DENV and WNV cases than in the non-severe disease and may present significant risk factors—together with advanced age—in complication of flavivirus infection.

Diabetes, hypertension, cardiac diseases and obesity are interrelated as they share similar cardiometabolic risk factors that result in the development of metabolic syndrome and the subsequent manifestation of this range of chronic diseases. These metabolic syndrome related diseases may impair the immune system to increase the level and duration of viremia [109, 139] and facilitate the passage of neurotropic flavivirus across the blood-brain barrier to predispose patients to neurologic complications [88, 118, 140]. Impairment of the innate immune system—that mediates the host defense to infection—render individuals more susceptible to a range of infectious diseases and severe illnesses [141–144]. In fact, the metabolic syndrome related chronic conditions are linked to endothelial dysfunction, attenuation of anti-inflammatory responses and a generation of a pro-inflammatory state; features that are also common in many infectious disorders [21, 141, 145]. For overproduction of pro-inflammatory cytokines such as ILs, TNF-α, IFN-γ and TGF-β is known to occur in severe DENV [146], WNV [147] and YFV [148] leading to cytokine storm and vasculopathy, hemorrhage, tissue damage and septic shock characteristic of severe flavivirus infections. Cytokine synthesis shift to the Th1 (microbicidal action of pro-inflammatory IFN-γ) from Th2 (anti-inflammatory IL-4, -10 and -13) in severe infection, when accompanied by the increased pro-inflammatory cytokine levels arising from chronic diseases, both can lead to endothelial dysfunction and a subsequent range of complications, including allergy, vascular leakage, ascites and pericardial effusion as observed in DENV [19, 141, 149–151]. In support, mononuclear cells from diabetic patients, when infected with DENV, produced significantly higher levels of IL-4, IL-10 and granulocyte-macrophage colony-stimulating factor compared to their healthy counterparts [152]. Conditions such hyperglycemia and cellular insulinopenia may also impair macrophage and lymphocyte functions leading to a status of reduced acquired immune response [143] that was linked to about 60% increased risk of pneumonia-related complications and hospitalization [153]. Further evidence for a possible role of altered innate immunity in mediating the association between metabolic syndrome-related comorbidities and severe clinical presentation of flavivirus infections can be also substantiated from the relatively high prevalence of asthma in severe DENV cases. Patients with asthma normally exhibit altered Th1 and Th2 responses [50, 59]. In general, although the etiological relationship between chronic comorbidities and severity of flavivirus diseases is yet to be fully elucidated, it may be reasonable to suggest that in infected patients, chronic conditions may synergistically attenuate both the innate and adaptive immune systems [154]. This may further impair critical components of immunity such as chemotaxis, phagocytosis, and the bactericidal activity of neutrophils and macrophages as well as downregulate the functions of T cells and neutrophils [142, 154] to exacerbate the complications of the infectious diseases.

Although the present study is the first to systematically report and quantify the prevalence of comorbidities in flavivirus infections, it has several limitations. It does not address the effect of flavivirus infection on the development of chronic comorbidities or attempting to substantiate a causality between the two conditions. Also, the study is not addressing the effect of integrated vector management practices on the infectious or chronic disease incidence or causal associations. We are simply reporting the prevalence of comorbidities in flavivirus severe and non-severe cases to warrant further studies investigating the effect of chronic comorbidities on the infectious disease outcome. The selected studies were mostly retrospective in nature with variable clinical and laboratory diagnostic criteria and control groups and were heterogeneous for exposures and outcomes. In the DENV studies, although the majority were conducted after the WHO adopted the new case classification of 2009 [120] to improve clinical management, many of the reports still used the WHO 1997 classification [121]. This absence of similar endpoint measures hindered the results comparability and may limit the generalizability of conclusions to other geographical regions or older age groups since the WHO 1997 criteria were based on disease patterns of children in Thailand [21, 121]. In addition to the retrospective nature of many of the identified studies, incomplete clinical datasets, relatively small sample size, inappropriate definition or selection of control population were noted as shortcomings in various reports leading to further limitations in interpreting the findings. In fact, most of the studies were hospital-based with a high potential for selection bias of appropriate control groups. Additionally, the studies retrieved in this review measured different outcomes for DENV (DHF and DSS—with different grades and definitions of severity) and WNV (WNE, WNM, poliomyelitis or acute flaccid paralysis) making results difficult to compare, broadening the scope of the outcome and rendering the findings challenging to extrapolate. Furthermore, the identified reports have shown several-fold of among-studies variance in the proportion of comorbidities which may have contributed to the significant heterogeneity observed in our report. Additional sources of heterogeneity may relate to the large among-studies variation in sample size and surveillance approaches. The heterogeneity of the selected studies was evident from the publication bias that may have been driven from the small-study effect, *i*.*e*., the possibility of including small studies with spuriously overstated estimates while discounting those without statistically significant effects that may have a lower possibility of being published. This was addressed, however, by stratifying the analysis by the flavivirus infection and including all studies related to the research question rather than excluding reports based on lack of quality. This may levy some limitations on the estimated contribution of comorbidities to severe flavivirus infections and render our results as a guide to generate more accurate estimates for national or international intervention strategies in subjects with comorbidities.

When assessing comorbidities, the studies showed that the various co-existing conditions have been either self-reported or record-based and did not allow for differentiating between those diagnosed before, after or during the infectious episodes. Furthermore, most of the retrieved reports did not provide clearly characterized, valid and reliably-defined comorbidities that were implemented consistently across studies. For example, the majority of the reports did not distinguish between the prevalence of type 1 and type 2 diabetes where both were combined despite their different characteristics, etiological factors and clinical features. Given this paucity of information, it was challenging to distinguish between the two types of diabetes for their role in viral diseases severity and complication. Furthermore, the lack of consistent reporting for the statuses of heart diseases made it difficult to evaluate the frequency of each heart condition. Therefore, we combined the different heart conditions under a single comorbidity. The utility of pooling the clinically different and heterogeneous pathologies of heart diseases may lead to losing significance of this observation and result in misinterpretation. This observation warrants developing more rigorous studies exploring the role of various heart condition in the severity of flaviviral infections. Given the possible extent of under-diagnosis for many of the assessed comorbidities, particularly in the developing world, misclassification of many co-existing medical conditions is likely substantial. Together with the possible underestimation for the frequency of infectious disease where not all asymptomatic cases will be detected, these factors may have led to lower estimates of prevalence for many of the assessed comorbidities. Lastly, since we identified only two studies for JEV and no studies met the inclusion criteria for YFV and ZIKV infections, the present report may be viewed as focusing primarily on DENV and WNV. This lack of available information on the prevalence of comorbidities in flavivirus diseases other than DENV and WNV, calls for developing large-scale studies to cover this knowledge gap.

In conclusion, the study of comorbidities in flavivirus infection is important for reducing the burden of the disease via guiding approaches for improved patient outcome or differential case management. We provided evidence for a higher prevalence of diabetes, hypertension, and heart diseases in severe cases of flavivirus infections such as DENV and WNV than in non-severe cases. These findings do not implicate causality between the chronic comorbidities and severe DENV or WNV. It simply demonstrates different profiles of comorbidities at different stages of the infectious diseases. Our results warrant further assessments to identify the nature and extent of the co-existence between comorbidities and infection. For example, standardized prospective case–control studies in regions of high infection prevalence would contribute to a better understanding for the etiological role of comorbidities in severe disease outcome when conducted with an agreed protocols of comorbidity assessments, infection classification, disease biomarker analysis and appropriate control groups. However, even in the absence of causal inference between the non-communicable and infectious diseases, it may be justified that once non-severe episodes of flavivirus infection are confirmed in subjects with comorbidities that they remain under close surveillance to avert complications. This may guide public health practitioners and clinicians to predict complications—at least partially—based on the presence of comorbidity. This can subsequently substantiate close observation, adequate treatment, or hospitalization following infection to avert severe disease outcome.

## Supporting information

S1 Table PRISMA Checklist for: Prevalence of chronic comorbidities in flavivirus infections: A systematic review and meta-analysis. (PDF)

S2 Table PICOST table for: Prevalence of chronic comorbidities in flavivirus infections: A systematic review and meta-analysis. (PDF)

S3 Table Specific search strategies for: Prevalence of chronic comorbidities in flavivirus infections: A systematic review and meta-analysis. (PDF)

S4 Table Publication bias analysis by Egger's regression intercept for: Prevalence of chronic comorbidities in flavivirus infections: A systematic review and meta-analysis. (PDF)

S1 Fig Funnel plot for systematic review for the prevalence of chronic comorbidities in flavivirus infections studies. Funnel plot is of standard error by logit evet ratio. The logit event rate for prevalence (horizontal axis) is presented against the standard error (SE) of the log of logit event rate (vertical axis) for dengue fever (panel a) and West Nile virus (panel b) studies. The SE inversely corresponds to the study size. Asymmetry of the plot can indicate publication bias. Open circles indicate the included studies. The plots show *t-value (P* for publication bias); Egger’s test.
(TIFF)
