# Supplementary material for: Prevalence of chronic comorbidities in dengue fever and West Nile virus: A systematic review and meta-analysis
Source: PLoS One. 2018 Jul 10;13(7):e0200200. doi: 10.1371/journal.pone.0200200 (PMC6039036; doi:10.1371/journal.pone.0200200)
Supplement: S2 Table — (PDF) [file pone.0200200.s002.pdf]

S2 Table: PICOST table.

Component	Criteria
**Population** (Flavivirus infection)	Individuals with a Flavivirus (both severe and non-severe cases), including the following:<br>– Dengue<br>– Yellow Fever<br>– West Nile<br>– Zika<br>– Japanese Encephalitis
**Intervention**	No intervention criteria
**Comparison**	– No comparison (if study does not specify the severity of flavivirus in the population)<br>– If applicable, severe Flavivirus to non-severe (infected but not severe) Flavivirus<br>– *Severity of flavivirus infection was classified as follows:*<br>○ Severe Dengue - any form of dengue that develops major complications such as dengue hemorrhagic fever (DHF grades I and II), dengue shock syndrome (DHF grades III and IV), fatal cases of dengue, dengue infections developing organ failures, such as acute renal failure and acute respiratory failure, clinically significant bleeding and/or requiring hospitalization in intensive care units.<br>○ Non-severe dengue also includes a study where "*Living; The controls were dengue- confirmed patients who recovered from the illness during their stay in the hospital and were subsequently discharged"<br>○ Severe West Nile - results in the development of West Nile neuroinvasive disease characterized by the development of encephalitis or meningitis or poliomyelitis or acute flaccid paralysis or death or by the patient staying in ICU or inpatient rehabilitation hospital; West Nile virus-associated retinopathy (WNVR); chorioretinitis
**Outcome** (Comorbidity/ Chronic Disease)	Prevalence of metabolic syndrome related conditions/chronic disease, including:<br>– diabetes<br>– hypertension<br>– Heart disease (cardiovascular disease, coronary artery disease (CAD), Coronary vascular disease, atrial fibrillation, chronic ischemic heart disease, acute coronary syndrome for duration <6 months, Cardiac Disorder, Cardiac Heart Failure, Congestive Cardiac Failure)<br>– stroke<br>– obesity<br>– asthma - does not include Chronic obstructive pulmonary disease (COPD)
**Study Design**	– Observational studies (including retrospective chart review)<br>– Case-control studies<br>– Randomized Control Trial (untreated arm)<br>– Cross-sectional studies
**Time**	– From inception of PubMed, Ovid MEDLINE(R), Embase and Embase Classic to the last week of November 2016<br>– The two most recent years of grey literature
**Outcomes not included**	– Cardiac complications/myocarditis<br>– Hypotension<br>– Circulatory failure<br>– Chronic renal failure<br>– Congestive cardiac failure<br>– Decompensated liver disease<br>– Cardiac arrhythmia
**Selection criteria for full text screening**	Inclusions:<br>– All patients need to have one or more of the following flaviviruses (Dengue, West Nile, Yellow Fever, Zika or Japanese Encephalitis).<br>– Study needs to report the frequency of at least one of the following outcomes: diabetes, hypertension, cardiovascular disease (CVD), coronary artery disease (CAD), stroke, obesity or asthma<br>Exclusions:<br>– Frequency of outcome (diabetes, hypertension, CVD, CAD, stroke, obesity or asthma) is not reported<br>– Entire study population has chronic disease condition and not flavivirus infection (e.g. people with diabetes where 100% of patients have Dengue)<br>– No relationship between chronic disease condition and flaviviral infection<br>– Duplicate study
