# Supplementary material for: Prevalence of chronic comorbidities in dengue fever and West Nile virus: A systematic review and meta-analysis
Source: PLoS One. 2018 Jul 10;13(7):e0200200. doi: 10.1371/journal.pone.0200200 (PMC6039036; doi:10.1371/journal.pone.0200200)
Supplement: S3 Table — (PDF) [file pone.0200200.s003.pdf]

S3 Table: Specific search strategies.

(1) Peer-Reviewed literature			
**Category**	**#**	**Searches**	**Results**
**MEDLINE(R)** Details: Ovid MEDLINE(R) 1946 to November Week 3 2016, Ovid MEDLINE(R) In-Process & Other Non-Indexed Citations inception to November 29, 2016			
Chronic Disease Terms	1	exp Diabetes Mellitus, Type 1/ or exp Diabetes Mellitus, Type 2/ or exp Diabetes Mellitus/	386574
	2	exp Hypertension/	250457
	3	exp Heart Diseases/	1047813
	4	exp Coronary Artery Disease/	52583
	5	exp Stroke/	115612
	6	exp Obesity/ or exp Obesity, Abdominal/ or exp Obesity, Morbid/	188394
	7	exp Hypertension/ or exp Obesity/ or exp Blood Pressure/ or exp Overweight/ or exp Coronary Disease/	826028
	8	1 or 2 or 3 or 4 or 5 or 6 or 7	2002032
Flavivirus Terms	9	exp Flavivirus/ or exp Flavivirus Infections/	31524
	10	dengue fever.mp. or exp Dengue/	11505
	11	exp West Nile Fever/ or exp West Nile virus/	5654
	12	exp Yellow Fever/	3096
	13	exp Zika Virus Infection/ or exp Zika Virus/ or zika.mp.	1730
	14	9 or 10 or 11 or 12 or 13	33649
Combination	15	8 and 14	209
De-duplication	16	remove duplicates from 15	194
Human limit	17	limit 16 to human	74
**EMBASE** Details: Embase Classic+Embase 1947 to 2016 Week 48			
Chronic Disease Terms	1	exp Diabetes Mellitus, Type 1/ or exp Diabetes Mellitus, Type 2/ or exp Diabetes Mellitus/	824799
	2	exp Hypertension/	650438
	3	exp Heart Diseases/	1689604
	4	exp Coronary Artery Disease/	290016
	5	exp Stroke/	164840
	6	exp Obesity/ or exp Obesity, Abdominal/ or exp Obesity, Morbid/	426405
	7	exp Hypertension/ or exp Obesity/ or exp Blood Pressure/ or exp Overweight/ or exp Coronary Disease/	1544221
	8	1 or 2 or 3 or 4 or 5 or 6 or 7	3357634
Flavivirus Terms	9	exp Flavivirus/ or exp Flavivirus Infections/	38338
	10	dengue fever.mp. or exp Dengue/	17944
	11	exp West Nile Fever/ or exp West Nile virus/	1765
	12	exp Yellow Fever/	4839
	13	exp Zika Virus Infection/ or exp Zika Virus/ or zika.mp.	1662
	14	9 or 10 or 11 or 12 or 13	38733

Combination	15	8 and 14	1476
De-duplication	16	remove duplicates from 15	1403
Human limit	17	limit 16 to human	1278

(2) Grey literature

Category	Terms	2016 Results	2015 Results	2014 Results[b,c]
**American Society of Tropical Medicine and Hygiene[a]** (2015-2016)				
Chronic Disease Terms	diabet*		11	
	hypertensi*		11	
	"blood pressure"		1	
	"heart disease"		4	
	cardiovascular		2	
	coronary		0	
	stroke		3	
	obes*		6	
	overweight		3	
	diabet* hypertensi* cardiovascular coronary stroke obes* overweight OR "blood pressure" OR "heart disease"		26	34
Flavivirus Terms	"yellow fever" only		37	
	"west nile" OR "yellow fever" OR dengue flavivir* Zika		320	377
Combinations[d]	chronic disease terms AND zika		0	
	chronic disease terms AND dengue		2	
	chronic disease terms AND "yellow fever"		0	0
	chronic disease terms AND "west nile"		0	0
	chronic disease terms AND dengue flavivir* Zika		2	5
**Open Forum Infectious Diseases - Infectious Diseases Society of America** (2014-2015)				
Chronic Disease Terms	diabet*	117		
	hypertensi*	34		
	blood AND pressure	14		
	heart AND disease	67		
	cardiovascular	53		
	coronary	10		
	stroke	5		
	obes*	33		
	overweight	5		

	diabet* or hypertensi* or cardiovascular or coronary or stroke or obes* or overweight or	184	
	diabet* or hypertensi* or cardiovascular or coronary or stroke or obes* or overweight or + (blood and pressure)	193	
	diabet* or hypertensi* or cardiovascular or coronary or stroke or obes* or overweight or + (blood and pressure) or (heart and disease)	231	
Flavivirus Terms	yellow and fever	1	
	(west and nile) OR (yellow and fever) OR dengue or flavivir* or Zika	52	
Combinations	((west and nile) OR (yellow and fever) OR dengue or flavivir* or Zika) AND (diabet* or hypertensi* or cardiovascular or coronary or stroke or obes* or overweight or (blood and pressure) or (heart and disease))	2	22
De-duplication	de-duplicated	2	20

[a]The two sources of grey literature were conference proceedings from the two most recent years from the American Society of Tropical Medicine and Hygiene and the Infectious Diseases Society of America.

[b]Included in EMBASE search.

[c]Results for 2014-2015 are combined in the "Open Forum Infectious Diseases - Infectious Diseases Society of America".

[d]Shaded cells form the complete set of combinations since there were character limits for each line.
