# Supplementary material for: Prevalence of chronic comorbidities in dengue fever and West Nile virus: A systematic review and meta-analysis
Source: PLoS One. 2018 Jul 10;13(7):e0200200. doi: 10.1371/journal.pone.0200200 (PMC6039036; doi:10.1371/journal.pone.0200200)
Supplement: S4 Table — (PDF) [file pone.0200200.s004.pdf]

S4 Table: Publication bias analysis by Egger's regression intercept.

Infection	Comorbidity	Egger's regression results			
		Intercept	S.E. (95% CI)	*t*-value	*p*
**Dengue fever**	Diabetes	0.691	2.56 (-4.52 - 5.91)	0.270	0.395
	Hypertension	3.980	2.075 -0.24 - 8.20)	1.918	0.032
	Heart diseases	-2.702	2.279 (-7.47 - 2.07)	1.185	0.125
	Asthma	0.669	1.47 (-2.72 - 4.06)	0.455	0.331
	Stroke	-1.985	2.165 (-7.10 - 3.13)	0.917	0.195
	Obesity	0.467	3.108 (-7.52 - 8.46)	0.150	0.443
**West Nile virus**	Diabetes	0.971	0.987 (-1.15 - 3.09)	0.983	0.171
	Hypertension	1.029	0.986 (-1.09 - 3.15)	1.043	0.157
	Heart diseases	2.443	1.14 (-0.15 - 5.04)	2.127	0.031
	Stroke	-0.054	1.763 (-5.66 - 5.56)	0.031	0.489
