# Supplementary figures and images for: Prevalence of chronic comorbidities in dengue fever and West Nile virus: A systematic review and meta-analysis
Source: PLoS One. 2018 Jul 10;13(7):e0200200. doi: 10.1371/journal.pone.0200200 (PMC6039036; doi:10.1371/journal.pone.0200200)

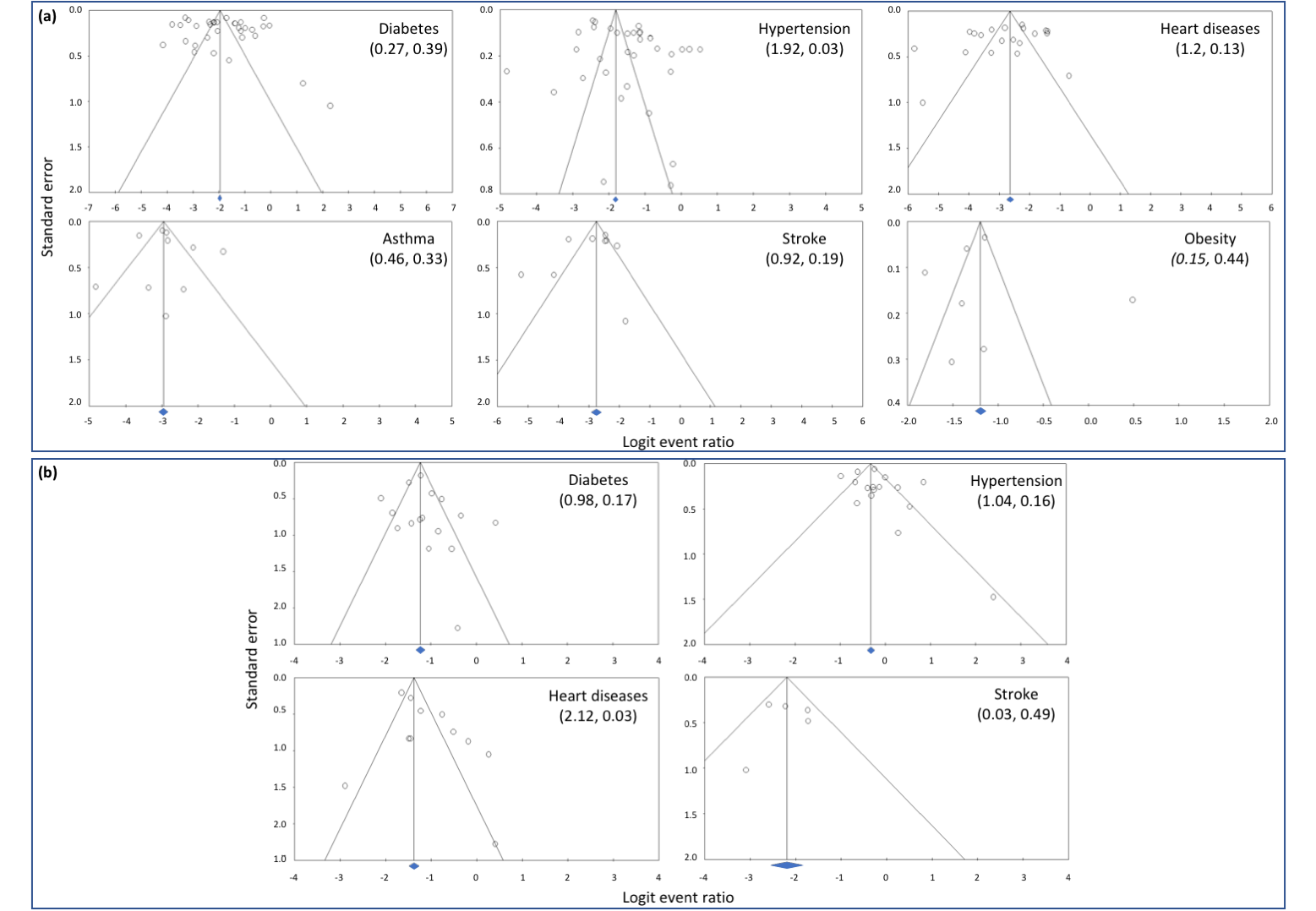

Supplement: S1 Fig — Funnel plot is of standard error by logit evet ratio. The logit event rate for prevalence (horizontal axis) is presented against the standard error (SE) of the log of logit event rate (vertical axis) for dengue fever (panel a) and West Nile virus (panel b) studies. The SE inversely corresponds to the study size. Asymmetry of the plot can indicate publication bias. Open circles indicate the included studies. The plots show t-value (P for publication bias); Egger’s test. (TIFF) [file pone.0200200.s005.tiff]
